# Global Brain Blood-Oxygen Level Responses to Autonomic Challenges in Obstructive Sleep Apnea

**DOI:** 10.1371/journal.pone.0105261

**Published:** 2014-08-28

**Authors:** Paul M. Macey, Rajesh Kumar, Jennifer A. Ogren, Mary A. Woo, Ronald M. Harper

**Affiliations:** 1 UCLA School of Nursing, University of California Los Angeles, Los Angeles, California, United States of America; 2 Brain Research Institute, University of California Los Angeles, Los Angeles, California, United States of America; 3 Department of Anesthesiology, David Geffen School of Medicine at UCLA, University of California Los Angeles, Los Angeles, California, United States of America; 4 Department of Radiological Sciences, David Geffen School of Medicine at UCLA, University of California Los Angeles, Los Angeles, California, United States of America; 5 Department of Neurobiology, David Geffen School of Medicine at UCLA, University of California Los Angeles, Los Angeles, California, United States of America; Max-Delbrück Center for Molecular Medicine (MDC), Germany

## Abstract

Obstructive sleep apnea (OSA) is accompanied by brain injury, perhaps resulting from apnea-related hypoxia or periods of impaired cerebral perfusion. Perfusion changes can be determined indirectly by evaluation of cerebral blood volume and oxygenation alterations, which can be measured rapidly and non-invasively with the global blood oxygen level dependent (BOLD) signal, a magnetic resonance imaging procedure. We assessed acute BOLD responses in OSA subjects to pressor challenges that elicit cerebral blood flow changes, using a two-group comparative design with healthy subjects as a reference. We separately assessed female and male patterns, since OSA characteristics and brain injury differ between sexes. We studied 94 subjects, 37 with newly-diagnosed, untreated OSA (6 female (age mean ± std: 52.1±8.1 yrs; apnea/hypopnea index [AHI]: 27.7±15.6 events/hr and 31 male 54.3±8.4 yrs; AHI: 37.4±19.6 events/hr), and 20 female (age 50.5±8.1 yrs) and 37 male (age 45.6±9.2 yrs) healthy control subjects. We measured brain BOLD responses every 2 s while subjects underwent cold pressor, hand grip, and Valsalva maneuver challenges. The global BOLD signal rapidly changed after the first 2 s of each challenge, and differed in magnitude between groups to two challenges (cold pressor, hand grip), but not to the Valsalva maneuver (repeated measures ANOVA, *p*<0.05). OSA females showed greater differences from males in response magnitude and pattern, relative to healthy counterparts. Cold pressor BOLD signal increases (mean ± adjusted standard error) at the 8 s peak were: OSA 0.14±0.08% vs. Control 0.31±0.06%, and hand grip at 6 s were: OSA 0.08±0.03% vs. Control at 0.30±0.02%. These findings, indicative of reduced cerebral blood flow changes to autonomic challenges in OSA, complement earlier reports of altered resting blood flow and reduced cerebral artery responsiveness. Females are more affected than males, an outcome which may contribute to the sex-specific brain injury in the syndrome.

## Introduction

Obstructive sleep apnea (OSA) is present in approximately 10% of the adult population, and is accompanied by symptoms related to central nervous system dysfunction, including excessive daytime sleepiness, high levels of depression and anxiety, elevated sympathetic tone, and memory deficits [1], [2]. These symptoms likely derive from changes to brain structure and function in the condition [3]–[10]. The causes of the brain impairments remain unclear; in addition to damaging effects of repeated hypoxic episodes, injury from impaired cerebral blood flow (CBF) regulation is a possibility. Ischemic conditions could arise from lower resting CBF [11]–[16], or from inadequate responses to changes in metabolic demand or physiological state, for example, the blood pressure changes elicited by standing or straining which are normally accompanied by CBF increases [17]. Weaker short-term CBF velocity responses to autonomic challenges appear in OSA in transcranial Doppler ultrasound recordings of limited cerebral arteries [18], [19], with measures taken external to the skull, but direct observations of responses within the brain tissue to acute challenges are lacking.

Rapid changes in CBF occur in response to blood pressure alterations. However, in subjects with OSA, the heart rate responses to blood pressure changes are time-lagged and weaker than in their healthy counterparts. In a companion paper, we showed that peripheral vascular responses to typical autonomic challenges (straining, temperature change) are impaired [20]. Early studies in OSA of autonomic responses to the Valsalva maneuver, a forced expiratory strain, showed reduced heart rate changes, which were interpreted as arising from impaired central nervous system regulation [9], [21]. Other challenges demonstrate similar patterns of reduced heart rate changes and altered neural responses in OSA [3], [6], [8]. Such autonomic impairments could contribute to cerebral injury. Brain tissue, in particular, is sensitive to acute periods of ischemia and rapid reperfusion [22], [23]; thus, a negative feedback cycle of brain injury resulting in impaired autonomic regulation, leading to inadequate vascular responses to daily demands, and further brain injury. However, the presence of impaired acute cerebral perfusion changes has only been shown in surface cerebral arteries, but not directly within brain tissue.

Rapid and non-invasive measures sensitive to changes in cerebral blood volume and oxygenation are available. Arterial spin labeling is a non-invasive magnetic resonance imaging (MRI) technique that enables absolute blood flow quantification, but at an effective time resolution of a minute or longer [24]. An indirect measure that can detect acute whole brain vascular changes is the global, whole-brain average of the blood oxygen level dependent (BOLD) magnetic resonance signal [25]. A BOLD signal, measured across the entire brain, can be recorded at a time resolution of less than two seconds with current technology, resulting in acquisition rates of 30 measurements per minute. The BOLD signal is sensitive to multiple phenomena, including levels of deoxyhemoglobin [25], which are related to blood oxygenation, CBF, cerebral metabolic rate of oxygen consumption (CMRO_2_), and cerebral blood volume (CBV) [26]–[28]. An increase in the concentration of deoxyhemoglobin, a paramagnetic substance, decreases the BOLD signal. Thus, a change in deoxyhemoglobin will be detected as a change in the BOLD signal from one measurement to the next, and the global (i.e., whole-brain) BOLD signal measured over time will reflect relative changes in deoxyhemoglobin concentration. Large CBF increases elevate the inflow of oxygenated blood and dilute deoxyhemoglobin, increasing the BOLD signal throughout the brain. Local changes in CBF, CMRO_2_, and CBV occur due to metabolic demands of neural activation, and can result in localized BOLD signal changes [25], [29]. However, during manipulations that grossly alter CBF [29]–[31], the influences of these localized effects on the whole-brain BOLD signal are minimal [32], and thus, changes in the global BOLD signal, i.e., the average signal value across the brain, provide a measure that principally reflects overall changes in volume and, to a lesser extent, oxygenation of blood.

To address the question of whether cerebral blood volume and oxygenation responses within brain tissue in OSA were impaired, we described whole-brain changes in global BOLD signal to transient blood pressure increases that normally elicit changes in cerebral blood flow and volume. The intention was not to mimic apneic stimuli, but to identify potential waking state impairments. We selected three autonomic stimuli that mimic daily challenges eliciting pressor responses, the cold pressor (cold temperature and pain or discomfort), the hand grip (muscle contraction), and the Valsalva maneuver, a forced expiratory effort that raises thoracic pressure (straining, lifting). These standard challenges elicit cerebral autoregulatory responses in healthy people [33]–[39], and impaired peripheral cardiovascular responses in OSA populations [20], [40], [41]. The objective was to assess the global BOLD signal during these three tasks, the cold pressor, hand grip, and Valsalva maneuver, in OSA and control populations, with additional separation by sex. Although not originally a focus of this investigation, the role of sex in brain and autonomic symptoms in OSA has emerged as an important factor [20], [42]. Thus, findings were also considered in females and males separately. We hypothesized that the BOLD changes in OSA would be muted and delayed, relative to controls. We further hypothesized that the pattern of impaired signals of OSA relative to controls would be similar in each of the sexes, but that systematic male-female differences would appear in BOLD responses.

## Methods

### Subjects

We recruited OSA patients from the UCLA Sleep Laboratory, and control subjects from the local community. No OSA subjects had started treatment for the sleep disorder, and all were recently-diagnosed. OSA patients were classified as either moderate based on an apnea/hypopnea index (AHI) between 15 and 29 events/hour, or severe based on an AHI>30 events/hour. No subjects were using psychotropic or cardiovascular medications, and none had a history of psychiatric disorders, cardiovascular disease, stroke, or other major illness. In particular, no subjects had a diagnosis of hypertension or atherosclerosis. Diabetes mellitus type 2 was confirmed in four patients and suspected in one. Scanner limitations precluded patients with metallic implants or weight over 125 kg. Subjects were classified as female or male according to their self-identified response on a screening questionnaire. All procedures were in accordance with the Declaration of Helsinki and approved by the UCLA Office of the Human Research Protection Program Medical Institutional Review Board 3, and subjects provided written, informed consent. These subjects overlap samples from other papers [5], [20], [42].

### Protocol

Subjects were asked to refrain from coffee and other substances with stimulants for the 24 hours prior to the study. After completion of screening and enrollment, subjects practiced the Valsalva maneuver and the hand grip challenges. The sequence of challenges was hand grip, Valsalva maneuver, and cold pressor. For the hand grip challenge, subjects were instructed to squeeze an inflated bag with the right hand. They were initially directed to briefly squeeze at maximum effort as a reference. The challenge consisted of a 16 s strain period (indicated by a blue light) at a subjective 80% of maximum. Subjects practiced this task outside, then inside the scanner. For the Valsalva maneuver, subjects were instructed to wait for a visual signal (blue light), then take a deep breath and expire from their chest (i.e., not from their oral cavity) until they achieved a pressure of 30 mmHg (indicated by a green light). Subjects were to maintain this pressure until the end of the 18 s challenge period (indicated by blue light being turned off), at which point they would breathe normally. Once subjects could perform the task, they were moved into the scanner, where they further practiced the challenge.

After practice, subjects were scanned with anatomical protocols, which allowed their physiologic functions to return to a baseline state. Following a minimum of 30 minutes of such scanning, the BOLD protocols were started, consisting of 436 s scans, including a 1 minute baseline, followed by four Valsalva challenges 1 minute apart, and a final 1 minute recovery. A similar pattern was followed for the hand grip challenge. The cold pressor challenge consisted of a 108 s baseline, 60 s foot immersion to approximately ankle height in cold water (4°C, verified by digital thermometer), and a 60 s recovery. Two investigators held the foot throughout the protocol, and lifted the foot into and out of the water at the appropriate times.

### Measurements: MRI and Physiologic Signals

We collected BOLD and anatomical scans with a 3.0 Tesla MRI scanner (Siemens Magnetom Tim-Trio, 8 channel head coil). The BOLD scans were collected with an echo-planar sequence in the axial plane [64×64 matrix size, 43 slices, no interslice gap, repetition time (TR) = 2000 ms, effective echo-time (TE) = 30 ms, FOV = 230×230 mm, slice thickness = 3.6 mm]. Two high-resolution, three-dimensional T1-weighted anatomical scans were collected sequentially using a magnetization-prepared-rapid-acquisition-gradient-echo pulse sequence (TR = 2200 ms; TE = 2.2 ms; inversion time = 900 ms; flip angle = 9°; matrix size = 256×256; FOV = 230×230 mm; slice thickness = 1.0 mm; number of slices = 176).

Physiological signals were recorded on a laptop with an a/d converter synchronized with the scanning signal. The expiratory pressure for the Valsalva maneuver was measured with a pressure sensor outside the scanner room, connected via a low-compliance tube into which a subject created expiratory pressure. Change in hand grip pressure was measured with a pressure sensor connected to a compressible bag which the subjects’ squeezed. This signal was not absolute; thus, only the presence or absence of pressure could be identified.

### Data Checking: Task Performance and Data Quality

The scans were checked for non-physiologic changes in signal intensity by plotting the global BOLD signal, and excluding series with step changes in intensity of more than 0.5%. Motion between scans of more than 4 mm in any direction, as calculated using the “Realign” procedure in SPM8 [43], was also cause for exclusion. The T1 anatomical scans were visually checked at the time of collection, and repeated if visible motion artifacts were observed.

Performance of each autonomic task was verified by inspecting the physiologic signals. For the Valsalva maneuver, subjects who did not achieve a sustained 30 mmHg pressure for the duration of each of the four challenges were excluded. The signals were checked to ensure all subjects exerted force during the four hand grip periods. Since the cold pressor was a passive challenge, no series were excluded based on performance.

### Analysis: Preprocessing

Preprocessing of the MRI images was performed using SPM8 and custom MATLAB software. Each subject’s two T1 anatomical scans were realigned and averaged, resulting in a higher signal-to-noise ratio relative to a single scan. Any series with motion greater than 4 mm in any direction, as identified by the SPM realignment procedure, was excluded. A brain mask was generated based on segmentation of the T1 image using the SPM procedures “unified segmentation” and “DARTEL” normalization [44], [45]. Voxels were classified as “brain” based on a combined probability of being gray or white matter of ≥0.5. The BOLD scans were realigned and linearly detrended to remove the typical gradual, but consistent, intensity increases in the scans (due to heating of the coils). The mean BOLD image was mapped to the T1 image, so that the brain mask derived from the high resolution scan could be applied to the BOLD sequence to exclude (mask) non-brain regions. The global BOLD signal was calculated based on the mean of each masked BOLD scan, thus representing signal within the brain.

### Analysis: Statistics

We used repeated measures ANOVA to assess global BOLD time trends in a mixed linear model implemented in SAS software (“proc mixed”) [46]. Random effects across subjects and groups were accounted for, and time was binned into baseline (single bin) and 2 s epochs corresponding to each scan. Overall model effects were tested, and according to the Tukey-Fisher criterion for multiple comparisons, only significant models and effects were assessed for within group (i.e., increases or decreases relative to baseline) or between group (i.e., differences in magnitude of response) signal differences at each time-point in the challenge and recovery. The analysis of each repeated task (Valsalva and hand grip) was performed on data grouped over the four challenges.

## Results

### Subjects

Detailed characteristics of the subjects are shown in Table 1. Of 110 subjects originally studied, 16 were excluded following data checking, resulting in a final sample of 94. Male subjects showed higher average severity of OSA than females, although only SAO_2_ nadir was significantly different.

**Table 1:** Subject information with group averages and standard deviations (± std).

	Female				Male				ANOVA
	Control		OSA		Control		OSA		*p* (F-test)
**N**	20		6		37		31		
**Age (years)**	50.5	±8.1	52.1	±8.1	45.6	±9.2	45.3	±8.4	0.06
**BMI (m^2^/kg)**	24.1	±5.3	32.4	±3.2	25.1	±2.8	30.2	±4.8	<0.001
*OSA Parameters*									
**AHI (events/hour)**	-		27.7	±15.6	-		37.4	±19.6	0.1
**SAO_2_ nadir**	-		86.0	±1.5	-		77.0	±9.2	0.03
**SAO_2_ baseline**	-		94.3	±1.5	-		94.8	±2.1	0.8

Group effects were tested with ANOVA (four-way for age and BMI, and two-way for OSA parameters).

**Figure 1:**
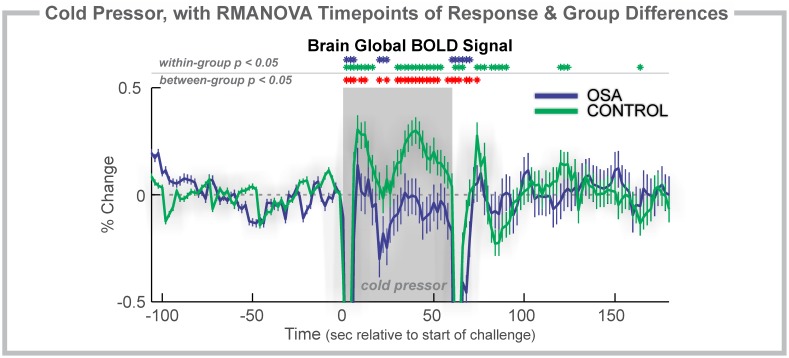
Cold pressor, 2 group. Time course of global BOLD signals during a cold pressor challenge (gray shaded rectangles). Change in BOLD signals, relative to baseline (group mean ± SE), with time-points of significant increase or decrease relative to baseline within-group, and time-points of between-group differences (RMANOVA, *p*<0.05).

**Figure 2:**
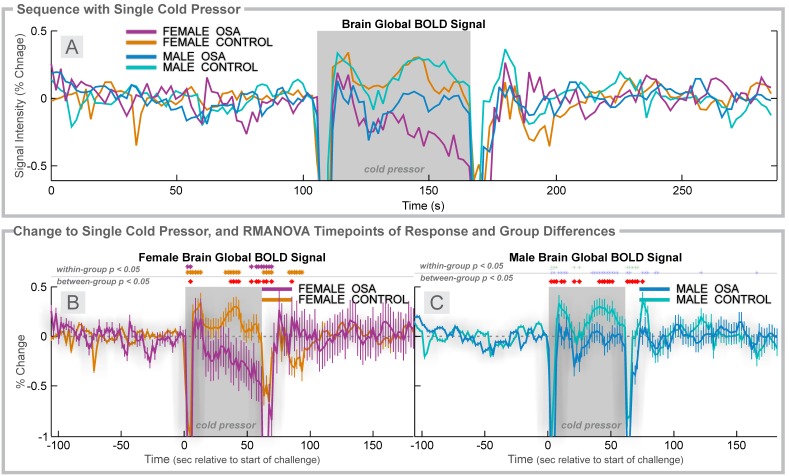
Cold pressor, 4 group. Time courses separated by sex of global BOLD signals during a cold pressor challenge (gray rectangles). **A:** Global BOLD signals in females (6 OSA, 20 control) and males (31 OSA, 37 control) during the complete sequence. For females (**B**) and males (**C**), changes in BOLD signals relative to baseline (group mean ± SE), with time-points of significant increase or decrease relative to baseline within-group, and time-points of between-group differences (RMANOVA, *p*<0.05).

**Figure 3:**
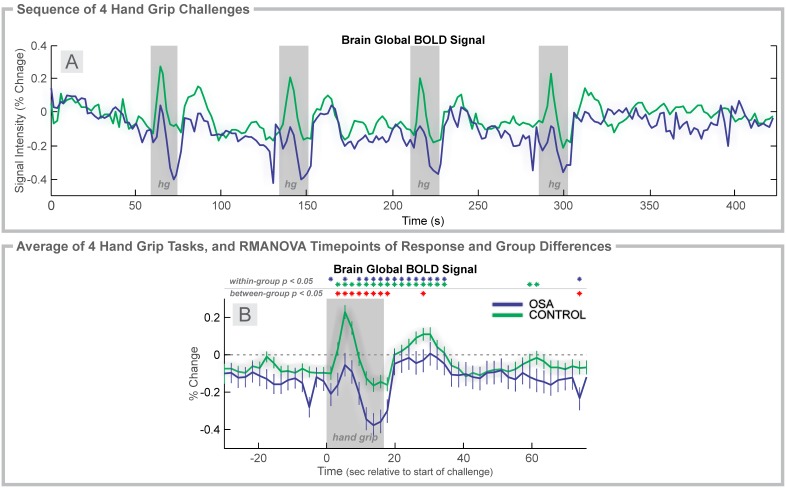
Hand grip, 2 group. Time course of global BOLD signals over four hand grip challenges (Grip effort; gray rectangles). **A:** Global BOLD signal in 37 OSA and 57 control subjects during the complete sequence. **B:** Average over four challenges of percent change in BOLD signals relative to baseline (group mean ± SE), with time-points of significant increase or decrease relative to baseline within-group, and time-points of between-group differences (RMANOVA, *p*<0.05).

**Figure 4:**
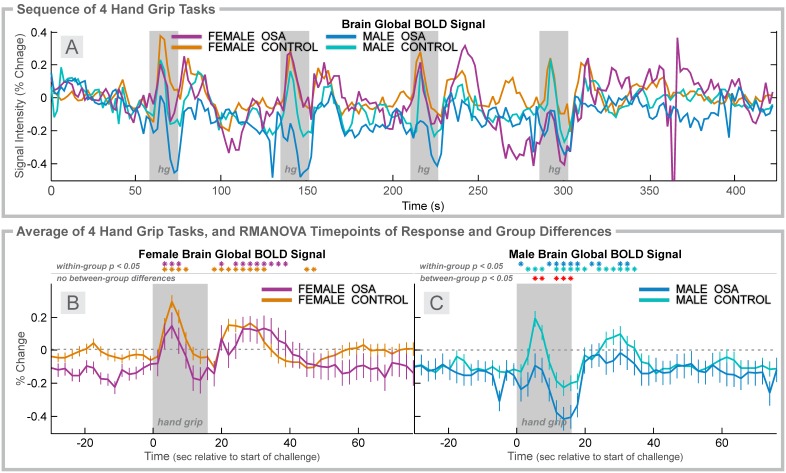
Hand grip, 4 group. Time courses, separated by sex, of global BOLD signals over four hand grip challenges (Grip effort; gray rectangles). **A:** Average global BOLD signal in females (6 OSA, 20 control) and males (31 OSA, 37 control) during the complete sequence. For females (**B**) and males (**C**), average over four challenges of change in global BOLD signals relative to baseline (group mean ± SE), with time-points of significant increase or decrease relative to baseline within-group, and time-points of between-group differences (RMANOVA, *p*<0.05).

**Figure 5:**
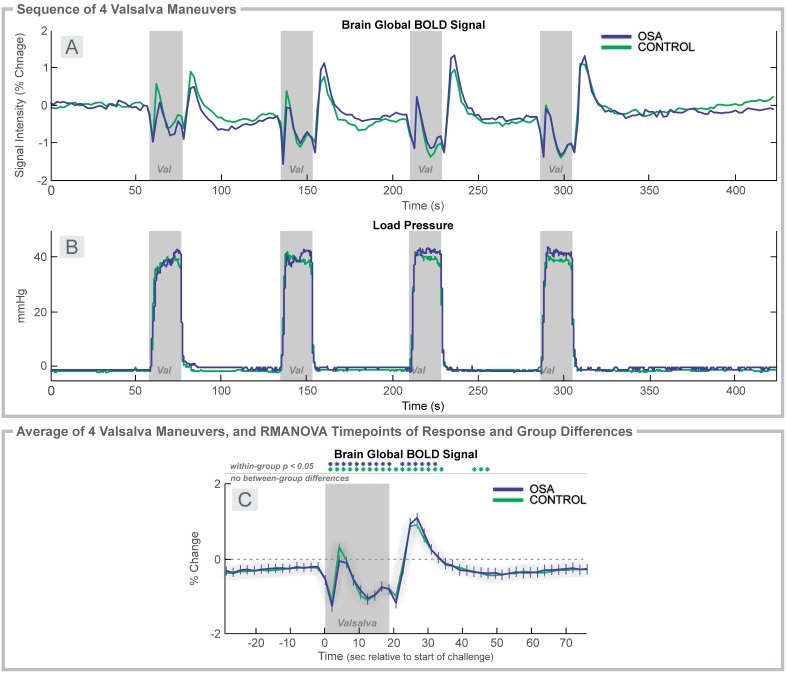
Valsalva, 2 group. Time course of global BOLD signals over four Valsalva maneuvers (Val; gray rectangles). **A:** Global BOLD signal, and **B:** expiratory pressure in 37 OSA and 57 control subjects during the complete sequence. **C:** Average over four challenges of change in BOLD signals relative to baseline (group mean ± SE), with time-points of significant increase or decrease relative to baseline within-group, and time-points of between-group differences (RMANOVA, *p*<0.05).

**Figure 6:**
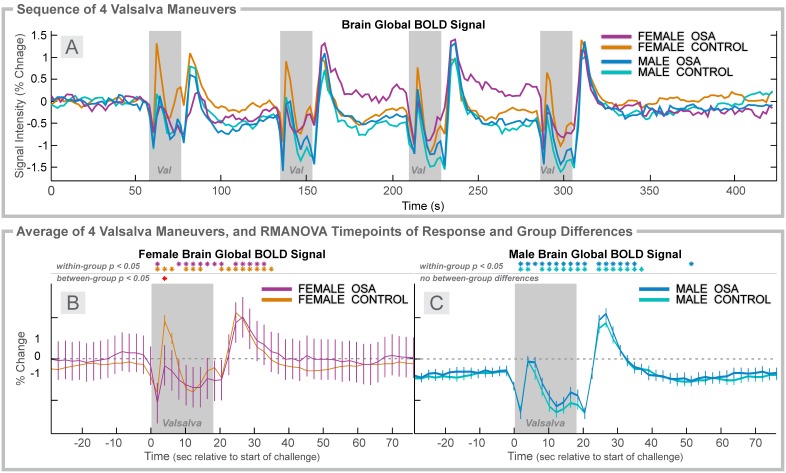
Valsalva, 4 group. Time courses, separated by sex of heart rate and global BOLD signals over four Valsalva maneuvers (Val; gray rectangles). **A:** Global BOLD signals in females (6 OSA, 20 control) and males (31 OSA, 37 control) during the complete sequence. For females (**B**) and males (**C**), global BOLD signal averages over four challenges of change relative to baseline (group mean ± SE), with time-points of significant increase or decrease relative to baseline within-group, and time-points of between-group differences (RMANOVA, *p*<0.05).

Time trends and time points of within- and between-group differences are shown for OSA and control groups in Figs 1–6, and corresponding raw data are in file S1 (cold pressor), S2 (hand grip), and S3 (Valsalva).

### Cold Pressor: Foot Movement Artifacts

Large signal changes, considered artifacts due to foot movement in and out of the cold water, were apparent in all subjects at the onset and termination of the challenge period (Figs. 1, 2). The movement lasted from 4 s to 6 s, with the BOLD signal showing large (>0.5%) declines immediately after insertion and removal from the water. The descriptions of results below exclude these periods, and address only signals considered reflective of changes in brain blood volume and oxygenation arising from the cold challenge per se. The outcomes are unlikely to have been systematically altered by the motion artifacts, as the recovery period showed signal values returning to baseline levels. If there had been a systematic shift caused by the movement, a difference in mean signals should have remained after the end of the tasks. If non-systematic, but large alterations that varied in direction across subjects had been present, the differences in the mean signals would have been masked, and statistical separation between groups not possible.

### Cold Pressor: OSA vs. Control

The global BOLD signal showed a rapid increase in both groups to a peak at 8 s (Fig. 1), but the magnitude of change was lower in OSA (0.14% vs. 0.31% in controls). The signal rapidly decreased to a minimum below baseline at time 20–22 s, to a lower value in OSA than control (−0.30% vs. −0.02%). For the remainder of the challenge, the signal increased, and then declined, but the OSA group remained at a lower magnitude than the controls. The signal returned to baseline 10–12 s after the challenge offset.

### Cold Pressor in Females

The global BOLD signal showed a slightly earlier and lower peak in female OSA than control subjects (0.19% at 8 s vs. 0.35 at 12 s; Fig. 2A, B). The control females showed higher global BOLD in the middle of the challenge period (32–42 s), whereas the OSA group showed declines, especially in the last 10 s. During recovery, the signals returned to similar values in both groups, although the control females showed below-baseline values 20–32 s into the recovery.

### Cold Pressor in Males

The global BOLD in OSA males increased to a lower peak than control males at time 8 s (0.13% vs. 0.34% in controls; Fig. 2A, C). Thereafter, both groups declined to baseline, but the control group showed a return to elevated values from 34–54 s into the challenge period, whereas the OSA males remained at baseline levels. During recovery, control males showed a transient increase (peaking at 0.37% at 14 s after the end of the challenge period); the OSA male global BOLD signal remained at baseline levels.

### Cold Pressor Variability

Standard deviations of responses to cold pressor application are shown for each time point as a measure of across-subject variability in File S1. Average values across all time points of the standard deviations are: OSA 0.60%; Control 0.56%; Female OSA 0.63%; Female Control 0.57%; Male OSA 0.59%; and Male Controls 0.57%.

### Hand grip: OSA vs. Control

The pattern of BOLD signal responses across the sequence of four challenges was consistent within each group, albeit with more variation in the OSA group (Fig. 3A). In contrast to the earlier-described heart rate values, the average (over challenges) global BOLD signal did not overlap between groups during the grip period (Fig. 3B). The OSA group showed sustained declines below baseline, whereas the control group signal increased to a peak at 6 s. The control group then showed a decline below baseline from 12 s, but the OSA group remained significantly lower (time 4–16 s of challenge). Both groups returned to baseline 20 s after the challenge.

### Hand Grip in Females

The pattern of BOLD signal responses across the sequence of four challenges was relatively consistent in the control group, but more variable in the OSA females (Fig. 4A). The average global BOLD signal in females showed a transient increase to a peak 6 s into the challenge, with a decline to baseline and rebound peak 12–16 s into the recovery (Fig. 4B). There were no significant group differences (the group×time effect was not significant; see Table 2).

**Table 2:** Statistics for mixed models.

	Overall Model		Effects					
			Group		Time		Group*Time	
	ChiSquare	*p*	F	*p*	F	*p*	F	*p*
*Cold Pressor*								
**All (mixed) ** **Fig. 1**	347	<0.0001	20	<0.0001	22	<0.0001	3	<0.0001
**Female ** **Fig. 4**	140	<0.0001	4	0.05	7	<0.0001	2160	<0.0001
**Male ** **Fig. 4**	201	<0.0001	17	0.0001	16	<0.0001	2	<0.0001
*Hand Grip*								
**All (mixed) ** **Fig. 2**	8192	<0.0001	3	0.1	17	<0.0001	4	<0.0001
**Female ** **Fig. 5**	952	<0.0001	2	0.2	6	<0.0001	1	0.1
**Male ** **Fig. 5**	6238	<0.0001	1	0.3	13	<0.0001	3	<0.0001
*Valsalva*								
**All (mixed) ** **Fig. 3**	7529	<0.0001	0	1	105	<0.0001	1	0.3
**Female ** **Fig. 6**	3261	<0.0001	0	1	20	<0.0001	3	<0.0001
**Male ** **Fig. 6**	4449	<0.0001	0.3	0.6	80	<0.0001	0.7	0.9

Chi Square statistics and *p* value are reported for repeated measures ANOVA, implemented as a mixed linear model with group, time and group by time as effects of interest (SAS proc mixed).

### Hand Grip in Males

The pattern of BOLD signal responses across the sequence of four challenges was most consistent in the control group, and moderately variable in the OSA males (Fig. 4A). The average (over challenges) global BOLD signal in males showed a transient increase to a peak 6 s into the challenge in both groups, but the peak was significantly lower in OSA than controls (0.04% vs. 0.30%; Fig. 4C). The signal was lower for most of the challenge in OSA vs. controls, but no group differences remained present during recovery.

### Hand Grip Variability

Standard deviations of responses to hand grip are shown for each time point as a measure of across-subject variability in File S2. Average values across all time points of the standard deviations are: OSA 0.50%; Control 0.43%; Female OSA 0.32%; Female Control 0.30%; Male OSA 0.53%; and Male Controls 0.50%.

### Valsalva Maneuver: OSA vs. Control

The BOLD signal responses across the sequence of four Valsalva maneuvers showed a consistent pattern, but with increasing magnitude from the first to last challenge, with similar time courses in both groups (Fig. 5A). The average (over challenges) global BOLD signal showed large, rapid changes in both groups, with no between-group differences (Fig. 5). Visualizing the sequence (Fig. 5A) shows that the one-minute recovery period was insufficient for the physiologic signals to return to baseline, which resulted in a below-zero baseline of the global BOLD signal over four averaged challenges (Fig. 5C). This variation was similar in both groups. The baseline and recovery periods in the average across challenges (e.g., Fig. 5B) show baseline values below 0 (0% represents the series baseline), suggesting that the deviation from baseline levels is consistent (i.e., a low global BOLD signal) across subjects. Visual inspection of the series timetrends (Figs 5A, 6A) supports the statistical findings of essentially no group differences (although one notable visual variation is the Female OSA group between the second and third challenges, Fig. 6A, this group was the smallest, and had high variability between subjects; Fig. 6B).

### Valsalva in Females

The BOLD signal responses across the sequence of four Valsalva maneuvers showed high variability in the female OSA group, but consistent patterns of similar amplitude in the control group (Fig. 6A). The average (over challenges) global BOLD signal in OSA females was highly variable (Fig. 6), with a significant group*time effect (Table 2), but with only one time-point of difference in the average signal. At 4 s into the challenge, a greater than 1% difference in signal was present between the groups, reflecting primarily a control increase.

### Valsalva in Males

The BOLD signal responses across the sequence of four Valsalva maneuvers were very similar in both groups, with a similar pattern, but of increasing magnitude from first to last challenge (Fig. 6A). The average (over challenges) global BOLD signals did not significantly differ between groups, and the male OSA group did not show the high variability of the female patients (Fig. 6). Of note, the global BOLD signal in males declined below baseline for the latter period of the challenge (phase II); whereas, in females this decline was not present. A drift due to lack of return to baseline in the 1 minute recovery periods is also most apparent in males rather than females (Fig. 6C, baseline/recovery periods).

### Valsalva Variability

Standard deviations of responses to the Valsalva maneuver are shown for each time point as a measure of across-subject variability in File S3. Average values across all time points of the standard deviations are: OSA 1.05%; Control 0.95%; Female OSA 1.01%; Female Control 0.78%; Male OSA 1.05%; and Male Controls 1.04%.

## Discussion

The amplitude of the global BOLD signal, indicative of brain blood volume and oxygenation, is altered in OSA patients relative to healthy control subjects during hand grip and cold pressor autonomic challenges, and the disruption differs by sex. Two unexpected findings emerged. Firstly, the OSA vs control amplitude differences did not appear for the Valsalva challenges. Secondly, in contrast with heart rate changes to these same three challenges [20], the OSA subjects did not show delayed timing of the global BOLD signal responses. Separation by sex highlighted differing female and male patterns in healthy control subjects, with greater OSA-related impairments in female vs male patients.

These findings suggest OSA is associated with a failure to adequately adapt cerebral perfusion to certain typical day-to-day challenges (e.g., gripping, temperature change), as mimicked by the standard tests used in this study. Presumably, the blood volume and oxygenation impairments would also be present during sleep, especially during obstructive apneas, which are accompanied by large, acute blood pressure changes [47], [48]. Impaired cerebral blood regulation would likely lead to acute periods of reduced tissue perfusion or oxygenation, which could be a potential source of the structural injury found in OSA [4], [5], [49].

The lack of a delay in the global BOLD responses in OSA relative to control subjects was surprising, given the timing differences in heart rate changes to the same challenges [20]. While the temporal resolution of the BOLD measurements may have masked shorter delays in cerebral vascular changes, some of the heart rate delays are over 2 s (temporal resolution of BOLD signal), thus the present findings show a lack of direct coupling between cerebral autoregulatory responses and heart rate. If the heart rate responses are driven by vagal and sympathetic regulatory brain areas, the data here suggest that cerebral autoregulatory mechanisms adapt more quickly than the autonomic regulatory pathways. (For review, see Winklewski 2013 [50].).

The brain global BOLD responses were lower to the foot cold pressor and hand grip challenges in OSA, relative to control subjects, but did not differ between groups to the Valsalva maneuver. The signal changes to the challenges principally reflect alterations in the volume of blood in the brain, with only modest influence of changes in blood oxygenation levels [32]. Thus, from the findings, we can assume that the cold pressor challenge elicits a modest initial peak in brain blood volume in the OSA group, but at a much lower magnitude than the control group. For the latter period of the challenge, the control group showed a sustained elevation of signal, whereas the OSA patients showed no increase, and even declines for a few seconds (<10 s). The OSA response to the hand grip was a weaker initial increase, followed by a similar decline in the global BOLD signal, such that the levels were lower throughout the challenge in the patient group. These OSA-control pattern differences contrast with the Valsalva responses, which showed similar large variations in both groups. The Valsalva maneuver elicits changes in arterial blood pressure from direct mechanical forces (thoracic pressure changes), and cerebral autoregulation during such blood pressure changes appears to be less impacted in OSA, relative to other types of pressor challenges.

If cerebral autoregulation operates similarly in all challenges, and the BOLD signal response is driven by blood pressure changes, the question that arises is whether the pressure changes in OSA were lower to the hand grip and cold pressor, but similar to the Valsalva maneuver, relative to the control group. Given the weaker heart rate changes in OSA to the hand grip and cold pressor, it is possible that blood pressure in the patient group does not increase as much [51], and hence, the observed smaller global BOLD response might simply reflect an appropriate autoregulatory response to a smaller change in pressure. However, the Valsalva maneuver also elicits a lower heart rate responses, so there is little evidence to support the possibility that lower BOLD signal changes in OSA are only related to lower blood pressure changes in that group.

One possible explanation for the muted responses in OSA is a ceiling effect, whereby a high resting cerebral blood volume in the patient group would limit the capacity for further transient increases. In theory, the higher blood gas CO_2_ levels typical in the sleep condition would be associated with vasodilation, and hence, larger vessel volume in OSA vs controls, but there is no evidence of such differences. On the other hand, OSA patients show lower resting CBF in several brain regions [11], [12], [16], [52], a variable closely related to brain blood volume. Furthermore, there is no reason to believe that a ceiling effect would be present during responses to hand grip and foot cold pressor, but not Valsalva challenges. If anything, the Valsalva challenge should have highlighted greater differences in the presence of a ceiling effect, since the magnitude of changes is much greater with that task, compared with the limb-initiated challenges. The combined evidence suggests that the present findings are unlikely to result from a ceiling effect.

The most notable difference in the global BOLD signal between sexes appeared in the cold pressor challenge, which was also reflected in very different heart rate responses in female vs. male OSA patients [20]. The cold pressor involves a combination of temperature and pain stimulation, and since females and males respond differently to objectively similar painful stimuli [53], [54], it is conceivable that the pain component contributes to those vascular differences. Sex differences in healthy populations appear in resting cerebral perfusion [55], [56]. Resting blood flow is higher in females [55], [56], which presumably is protective against ischemic conditions. However, whether this female advantage is also present in OSA is unknown. Acute blood flow responses in female patients appear not to be advantageous compared to those in males, in the light of both the weaker global BOLD responses reported here, and greater brain injury in OSA females [42].

Cerebral blood flow, measured indirectly here with the global BOLD signal, is a function of blood pressure and cerebral vascular resistance, factors regulated by chemical, hormonal, mechanical, and neural factors [57], [58]. The Valsalva maneuver activates thoracic, oral, and vascular pressure receptors, and includes a central voluntary effort component. Those elements did not affect the global BOLD signal in OSA, in spite of greatly muted heart rate responses [20]. The similar Valsalva BOLD responses in OSA and controls, and equivalent return to baseline in the other challenges, suggest that CBF changes to some pressure stimuli are relatively less impacted in OSA than to other demands. The findings from the other challenges suggest that specific aspects of the hand effort and cold sensory stimuli, possibly the central elements mediating those components, exert essential roles in modulating the BOLD response.

Whether physiology during these awake challenges parallels the cerebrovascular patterns during obstructive events in sleep is unknown, given the state-dependence of cerebral hemodynamics [48], [59]–[61]. The impaired reactivity found in OSA patients during wakefulness may be exacerbated during sleep [62], and especially in REM sleep, where substantial reorganization of vascular control and chemoreception occurs relative to non-REM states [63]–[65]. Further changes in hemodynamic organization may also occur during apneic episodes [59], [66]–[68], and there is evidence that reduced CBF can contribute to breathing instability during sleep, including the occurrence of apneas [69], [70]. Finally, the dysfunction observed here in the awake state may differ from that found in particular sleep states [71], [72].

Impaired perfusion may contribute to the structural brain injury found in OSA, especially during the large blood pressure changes associated with obstructive events. Cerebral blood volume dips after obstructive events [73], presumably reducing perfusion below optimal levels. Indirect evidence using near-infrared spectroscopy suggests that CBF is insufficiently increased to compensate for the rapid blood pressure changes during obstructive events [48]. Many of the brain areas affected in OSA have a greater oxygen demand in response to challenges, based on observations in healthy subjects [74], [75]. While lower perfusion does not necessarily mean lower oxygen delivery, the damage found in OSA, particularly in limbic regions mediating pressor challenges, may result from insufficient perfusion at a time of the greater demand specific to those areas.

### Limitations

The hand grip was a subjective, non-isometric challenge, whereas ideally, the protocol would involve sustaining a grip pressure at a predetermined, quantified percentage of measured, as opposed to perceived, maximum. Thus, unlike the Valsalva and cold pressor challenges, the hand grip effort may not be considered a strictly equivalent challenge across subjects or groups. However, the timing and pattern of physiologic responses were consistent across subjects within groups. The generalizability of the sex-specific findings is limited, since the female OSA group had only six subjects. No delay was found in the BOLD responses in OSA to any challenge, in contrast with the slowed heart rate changes (1–2 s late); however, the time resolution for the BOLD signal was 2 s, whereas the heart rate was 1 s, so it is possible that short delays (<2 s) were also present in the cerebral responses. The BOLD signal is an average of signals over the whole brain, but regional variations may exist. Thus, for example, the absence of differences between OSA and control subjects to the Valsalva maneuver could potentially result from masking by increases in some regions canceling declines in others. The confounding effects of changes in oxygen consumption due to neural activation (or deactivation) are likely modest, since neural effects are local, whereas the global BOLD signal is measured across all brain tissue. Caution should be used when interpreting a lower global BOLD signal as reflecting lower oxygen delivery or consumption, since changes in CBF and perfusion are possible without changes in metabolism due to compensatory changes in oxygen extraction.

Possible subject classification confounds include the presence of diabetes in four (likely five) OSA patients, as well as the potential for undiagnosed hypertension, with both conditions associated with neural deficits [76], [77] and altered cerebral blood flow regulation in hypertension especially [78]–[80], and to some stimuli in diabetes mellitus type 2 [81]–[83]. The OSA and control groups were not matched for BMI, although the extent to which obesity influences cerebral perfusion is unclear [84].

## Conclusions

Whole brain blood volume and oxygenation responses to hand grip and cold pressor autonomic challenges are blunted in OSA patients; these differences are exacerbated in female subjects. The brain vascular responses, likely reflecting changes in CBF to various stimuli, are less affected in OSA by the stimuli associated with the Valsalva maneuver than by cold and hand grip challenges. The exacerbation of impairments in female vs. male OSA may contribute to the additional brain injury found in female patients.

## Supporting Information

File S1 Cold pressor global BOLD raw data, in Excel format. Individual subject values are in worksheets specific to sleep status and sex (CONFEMALE, CONMALE, OSAFEMALE, OSAMALE), and group means (Y) and standard deviations (STDEV(Y)) are in the “Group Means” worksheet. Values are recorded every 2 s (“X” column in “Group Means” worksheet).
(XLSX)

File S2 Hand grip global BOLD raw data, in Excel format. Individual subject values are in worksheets specific to sleep status and sex (CONFEMALE, CONMALE, OSAFEMALE, OSAMALE), and group means (Y) and standard deviations (STDEV(Y)) are in the “Group Means” worksheet. Values are recorded every 2 s (“X” column in “Group Means” worksheet).
(XLSX)

File S3 Valsalva global BOLD raw data, in Excel format. Individual subject values are in worksheets specific to sleep status and sex (CONFEMALE, CONMALE, OSAFEMALE, OSAMALE), and group means (Y) and standard deviations (STDEV(Y)) are in the “Group Means” worksheet. Values are recorded every 2 s (“X” column in “Group Means” worksheet).
(XLSX)
